# Electrophoretic Determination of L-Carnosine in Health Supplements Using an Integrated Lab-on-a-Chip Platform with Contactless Conductivity Detection

**DOI:** 10.3390/ijms241914705

**Published:** 2023-09-28

**Authors:** Iva Pukleš, Csilla Páger, Nikola Sakač, Bojan Šarkanj, Brunislav Matasović, Mirela Samardžić, Mateja Budetić, Dean Marković, Marija Jozanović

**Affiliations:** 1 Department of Chemistry, Josip Juraj Strossmayer University of Osijek, Cara Hadrijana 8, 31000 Osijek, Croatia; jcf9b6@pte.hu (I.P.); brunislav.matasovic@kemija.unios.hr (B.M.); mirelas@kemija.unios.hr (M.S.); mbudetic@kemija.unios.hr (M.B.); 2 Doctoral School of Chemistry, University of Pécs, Ifjúság útja, 7624 Pécs, Hungary; 3 Department of Analytical and Environmental Chemistry, Faculty of Sciences, University of Pécs, Ifjúság Útja, 7624 Pécs, Hungary; 4 Institute of Bioanalysis, Medical School, Szentágothai Research Center, University of Pécs, Honvéd Utca 1, 7624 Pécs, Hungary; csilla.pager@aok.pte.hu; 5 Faculty of Geotechnical Engineering, University of Zagreb, Hallerova 7, 42000 Varaždin, Croatia; 6 Department of Food Technology, University North, Trg dr. Žarka Dolinara 1, 48000 Koprivnica, Croatia; bsarkanj@unin.hr; 7 Scientific Center of Excellence for Personalized Health Care, Josip Juraj Strossmayer University of Osijek, Trg Svetog Trojstva 3, 31000 Osijek, Croatia; 8 Department of Biotechnology, University of Rijeka, Radmile Matejčić 2, 51000 Rijeka, Croatia; dean.markovic@biotech.uniri.hr

**Keywords:** L-carnosine, capacitively coupled contactless conductivity detection, green analytical chemistry, lab-on-a-chip, microchip electrophoresis

## Abstract

The health supplement industry is one of the fastest growing industries in the world, but there is a lack of suitable analytical methods for the determination of active compounds in health supplements such as peptides. The present work describes an implementation of contactless conductivity detection on microchip technology as a new strategy for the electrophoretic determination of L-carnosine in complex health supplement formulations without pre-concentration and derivatization steps. The best results were obtained in the case of +1.00 kV applied for 20 s for injection and +2.75 kV applied for 260 s for the separation step. Under the selected conditions, a linear detector response of 5 × 10^−6^ to 5 × 10^−5^ M was achieved. L-carnosine retention time was 61 s. The excellent reproducibility of both migration time and detector response confirmed the high precision of the method. The applicability of the method was demonstrated by the determination of L-carnosine in three different samples of health supplements. The recoveries ranged from 91 to 105%. Subsequent analysis of the samples by CE-UV-VIS and HPLC-DAD confirmed the accuracy of the obtained results.

## 1. Introduction

The dipeptide L-carnosine is a vital biomolecule due to its contribution to numerous regular activities in the excitable tissues of vertebrates. The production of carnosine occurs in the liver through the condensation of amino acids β-alanine and L-histidine. In the human diet, exogenous L-carnosine is consumed through the consumption of foods of animal origin, mainly meat. The L-carnosine content of meat varies considerably between different animal species and different meat parts. Therefore, the amount of exogenous L-carnosine depends primarily on personal dietary choices [1,2].

The health benefits of L-carnosine to prevent and treat various medical conditions have been demonstrated in a large number of studies. It has been shown that L-carnosine has potent antioxidant and buffering effects, as well as strong chelating and neuroprotective properties [3,4,5]. L-carnosine treatment can regulate blood glucose levels and prevent drug-induced cardiotoxicity and neurotoxicity [6]. Moreover, L-carnosine showed beneficial effects on neurodegenerative diseases such as Alzheimer’s disease [7,8]. Due to all the above listed beneficial properties, L-carnosine is widely used in the form of health supplements. In addition to its health purposes, L-carnosine containing health supplements are also used by athletes for the enhancement of ergogenic effects during intense training. Polaperzinc is a chelated form of zinc and L-carnosine. It is an approved zinc-containing drug used to clinically treat gastric ulcers and to reduce toxicity caused by radiation therapy [9].

To avoid the adverse effects of taking supplements, the recommended daily dose should be specified, and labels should indicate the exact amount of active ingredients. For instance, excessive blood pressure lowering may occur if L-carnosine is taken at the same time as medications to lower blood pressure. Consequently, poor quality control and deficient legal regulation that is trotting behind the commercial boom of the health supplements market may lead to potential health problems for consumers or the ingestion of an ineffective dose. Therefore, the need for the development of reliable and standardized analytical methods for health supplements has been widely recognized.

Existing methods for the determination of small peptides, although accurate and precise, are often complex, expensive, and require a high consumption of toxic organic solvents. Currently, high-performance liquid chromatography (HPLC) with integrated fluorescent [10] or mass spectrometry (MS) [11] detection is the most widely used method for the peptide analysis of foods. Ultraviolet (UV) detection can also be employed, but dipeptides have low UV absorbance, which requires a time-consuming derivatization process. Gatti et al. reported a reverse-phase HPLC-UV method for the analysis of L-carnosine in health supplements that employs the tedious derivatization step with 2,4-dinitrofluorobenzene [12]. Furthermore, nuclear magnetic resonance spectroscopy (NMR) was used to determine L-carnosine in foods [13]. Capillary electrophoresis (CE) is a green alternative to HPLC that can also be used for the routine analysis of pharmaceutical and biopharmaceutical substances. The main advantages of CE are the higher separation efficiency and the absence of toxic organic solvents. Some reports describe the use of CE for the analysis of amino acids and dipeptides in health supplements, but to our knowledge, there is no publication on the analysis of L-carnosine in health supplements [14,15,16]. The available studies report the use of mass spectrometry [17], amperometry [18], laser-induced fluorescence [19], and UV detection [20] for the CE analysis of L-carnosine in various biological and food samples. Microchip electrophoresis (MCE), as a miniaturized form of CE, is an even more environmentally friendly method that allows for a short separation time and the use of less expensive equipment. Zhao et al. reported a highly sensitive MCE method for the determination of L-carnosine and related peptides in biological samples. To convert the species to a detectable form, they used a time-consuming chemiluminescence reaction between hydrogen peroxide and N-(4-aminobutyl)-N-ethylisoluminol-labelled peptides in the presence of adenine and Co^2+^ [21]. Fresta et al. reported the use of fluorogenic reagent naphthalene-2,3-dicarboxaldehyde to quantify intracellular carnosine in macrophage cell lysates [22], and Chen et al. analyzed 4-fluoro-7-nitro-2,1,3-benzoxadiazole labeled L-carnosine and niacinamide in cosmetics [23]. Additional simplification of MCE analysis was achieved by integrated capacitively coupled contactless conductivity detection (C^4^D). Until now, MCE-C^4^D analysis was reported for diverse analytes, moreover, Tůma et al. summarized its usage in clinical samples, pharmaceuticals, drinking water, beverages, and foodstuffs [24,25]. Jozanović et al. reported the use of homemade MCE-C^4^D devices for the simultaneous determination of L-carnosine and L-anserine in meat samples. The study demonstrates the suitability of C^4^D for microfluidic analysis, considering portable devices and particularly low manufacturing costs. The major contribution of C^4^D is its ability to analyze samples with complex compositions [26,27]. Difficulties in the analysis of health supplements usually emerge due to the complex and non-specific composition of health supplements, which often contain various adulterants and different herbal extracts in addition to excipients. The herbal extract contents are usually listed only in percentages on the declaration, but the composition of the extract is not indicated. Such, unknown and possibly interfering components can be a particular problem in analysis, especially in optical detection. This report describes the development of a method for the determination of L-carnosine in health supplements using microchip electrophoresis. The objective was to develop a low-cost, rapid, simple, portable, and environmentally friendly method for the analysis of health supplements. Therefore, the development of the method was focused on avoiding the derivatization of the analyte. For this purpose, the MCE technology was conjugated with the C^4^D system. The results obtained with the developed method were compared with the results obtained with CE-UV-VIS and HPLC conjugated with a diode array detector (DAD).

## 2. Results and Discussion

### 2.1. Method Optimization

**Figure 1:**
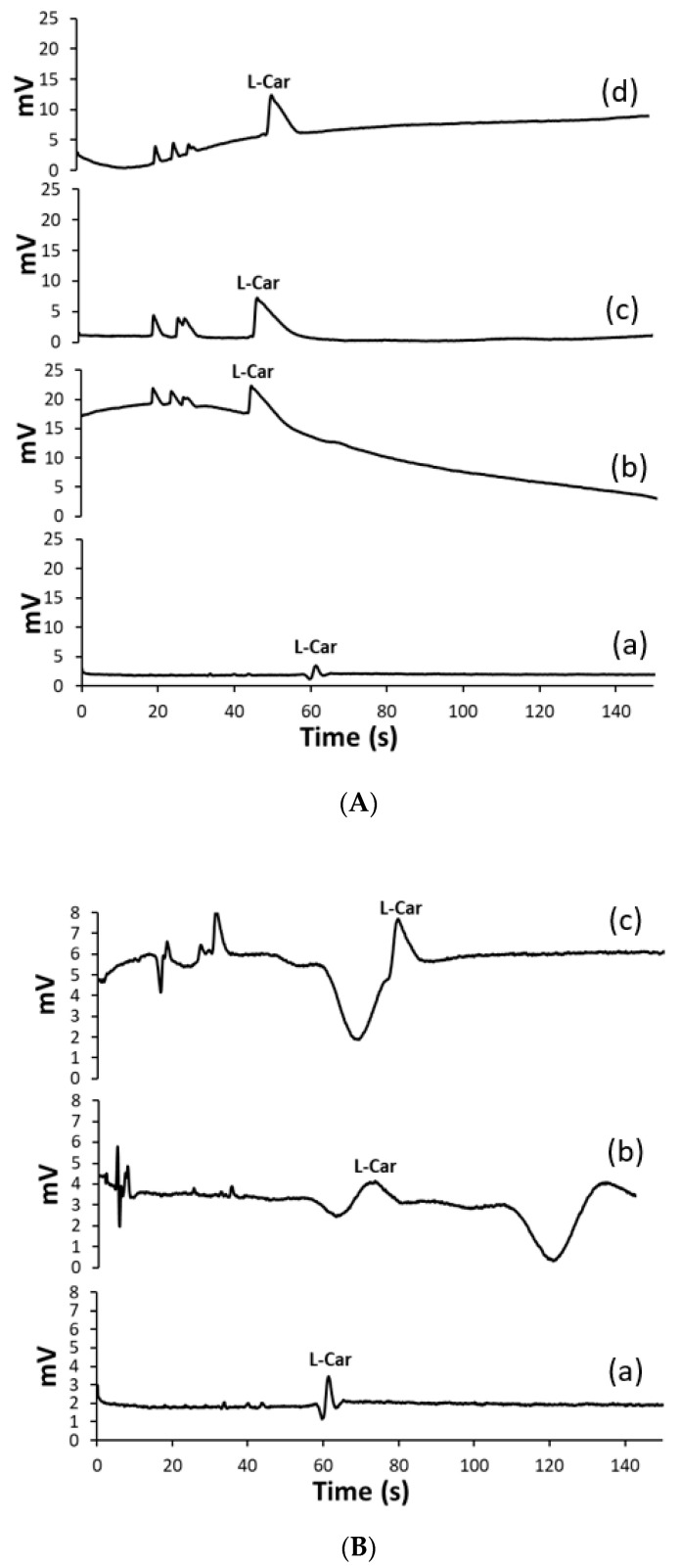
The MCE-C^4^D electropherograms of 5 × 10^−5^ M L-carnosine standard solutions in (**A**) (**a**) 0.5 M Hac (pH 2.52), (**b**) 7.5 × 10^−4^ iminodiacetic acid (pH 2.5), (**c**) 2.4 × 10^−9^ M tartaric acid (pH 5.8), and (**d**) 1.41 × 10^−3^ M citric acid (pH 3); (**B**) (**a**) 0.5 M HAc (pH 2.52), (**b**) 0.01 M 2-hydroxyisobutyric acid + 2 × 10^−4^ M sodium acetate + 0.4 M acetic acid (pH 2.36), and (**c**) 0.01 M 2-hydroxyisobutyric acid + 2 × 10^−4^ M rubidium acetate + 0.4 M HAc (pH 2.36).

**Figure 2:**
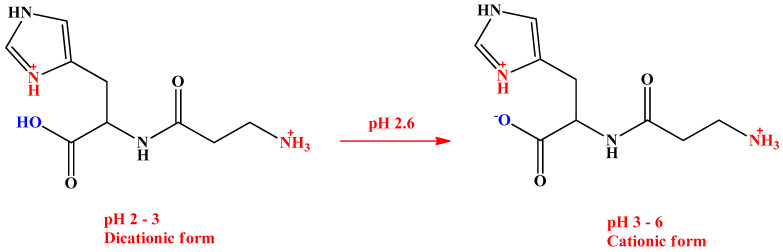
Schematic layout of ionic forms of L-carnosine in acidic media.

The response of the detector to L-carnosine was studied to determine the optimal operating frequency, amplitude, and excitation voltage, using a buffer solution consisting of 0.5 M acetic acid (HAc). Frequency studies ranged from 250 to 800 Hz and the amplitude from 10 to 60%. The best detectability was achieved by applying an excitation sine wave of 400 kHz with an amplitude of 40%. These parameters were used for subsequent optimization of the electrophoretic measurement. In order to use a BGE characterized by a sufficiently large difference in conductivity and as similar in mobility as possible with respect to the analyte, a variety of different BGE were tested in this study. The MCE system had a normal polarity, and accordingly, an acidic pH was considered in the selection of the BGE to ensure the cationic form of L-carnosine and its migration to the cathode. Therefore, the following organic acids and their mixtures were tested: 0.5 M HAc (pH 2.52), 7.5 × 10^−4^ M iminodiacetic acid (pH 2.5), 2.4 × 10^−9^ M tartaric acid (pH 5.8), 1.41 × 10^−3^ M citric acid (pH 3), 0.01 M 2-hydroxyisobutyric acid + 2 × 10^−4^ M sodium acetate + 0.4 M HAc (pH 2.36), and 0.01 M 2-hydroxyisobutyric acid + 2 × 10^−4^ M rubidium acetate + 0.4 M HAc (pH 2.36). Baseline drift can be observed in Figure 1A for curves (b) and (c), which correspond to BGE consisting of iminodiacetic acid and citric acid, respectively. It is assumed that the high conductivity of these acids contributed to the generating Joule heating and therefore caused unstable trends. Hence, these acids were excluded from further research in BGE optimization. Using BGE consisting of tartaric acid, presented with curves (c), resulted in board and unsymmetrical peaks, therefore they were also eliminated from further investigation. Only curve (a), which represents HAc, shows a stable trend, and symmetrical and sharp peaks, thus it was chosen for further investigation. The mixtures containing HAc, its conjugated salts, and 2-hydroxyisobutyric acid were further tested. These systems also did not yield useful results, as can be seen from the electropherograms shown in Figure 1B(b,c). The best results were obtained with 0.5 M HAc (Figure 1B(a)), which can be explained by the fact that it had the lowest conductivity and consequently the background noise was minimized. In this way, more intense peaks and a better readable signal were obtained. The suitability of BGE consisting of HAc for C^4^D detection of AAs and related biomolecules is also described by Peter Tůma [24]. In this study, the BGE pH 2.52 caused complete protonation of the L-carnosine molecule, the β-alanine amino group, and the L-histidine imidazole ring, as well as protonation of the L-histidine carboxyl group. Under these conditions, the dominant form of L-carnosine was charged with +2 and therefore migrated to the cathode under the influence of the electric field (Figure 2). The pK_a_ of the L-histidine carboxyl group in L-carnosine is 2.64, which means that faster migration of L-carnosine is achieved if the pH of the BGE is lowered below this value, resulting in sharp and high peaks. Thus, the use of the proposed BGE ensures high ion-mobility and peak symmetry, so further lowering of the pH was not necessary. Moreover, very acidic media could cause serious damage to polymer chips such as PMMA chips.

In order to achieve fast and efficient separation, another focus of the study was to optimize the injection and separation parameters. The influence of injection voltage was tested with a 0.20 kV steps increment in the range of +1.00 to +1.50 kV. The most symmetrical peak shape was obtained at an applied injection voltage of +1.00 kV. Injection times were evaluated from 12 to 25 s, with peak broadening being noted at times greater than 20 s. Moreover, lower reproducibility was recorded for injection times greater than 20 s. As a result, a 20 s injection time and +1.00 kV injection voltage were selected for further method development. The separation voltage was evaluated in the range from +2.00 to +2.75 kV, in 0.20 kV steps. A value of +2.75 kV was chosen due to the rapid analysis and maximum peak area. Since the migration time for L-carnosine was 61 ± 0.42 s, separation windows of 120 s, 180 s, and 240 s were tested. Baseline drift occurred when a separation time of 120 or 180 s was applied, so a separation time of 240 s was used in the experiments.

### 2.2. Analytical Features of the Method

The statistical parameters used to evaluate the method are summarized in Table 1. They represent the quality and consistency of the results in terms of the repeatability and mean precision of the data, as well as the sensitivity and linearity achieved. In this study, the linearity of the detector response was determined from the peak height calibration curve, which showed more analytically acceptable results than the peak area curve. For L-carnosine standard solutions ranging from 5 × 10^−6^ to 5 × 10^−5^ M, the standard deviation (SD) and relative standard deviation (RSD) of peak height varied from 1.3% to 12%, and from 3% to 19%, respectively. For the peak area, the values ranged from 4% to 63%, and from 4% to 51%, respectively. In the tested concentration range of 5 × 10^−5^ to 5 × 10^−6^ M, the correlation coefficient of 0.9976 indicated a good linear behavior of the C^4^D response to L-carnosine. Therefore, the analytical dependence of peak height on L-carnosine concentration was considered suitable to achieve satisfactory stability of the detector response. In this study, an achieved L-carnosine detection limit (LOD) of 2.5 × 10^−6^ M was obtained experimentally, which is comparable to the previously reported values [26,27]. The experimental limit of quantification (LOQ) was 5 × 10^−6^ M, thus allowing for reliable quantification of L-carnosine in commercial health supplements. LOD and LOQ were also determined by analyzing different solutions of L-carnosine and measuring the signal-to-noise ratio. The limit of detection (LOD) was the concentration giving a signal-to-noise ratio of 3:1, and the limit of quantitation (LOQ) was the concentration giving a signal-to-noise ratio of about 10:1, with an RSD of less than 10% with triplicate analysis. Thus, the calculated LOD and LOQ are 0.12, and 0.39 µM, respectively. The repeatability and reproducibility of the migration time were calculated by using five series of five consecutive injections. RSD values ranged from 0.5% to 2%, confirming the excellent repeatability and reproducibility of the method.

### 2.3. Determination of L-Carnosine in Real Samples

**Figure 3:**
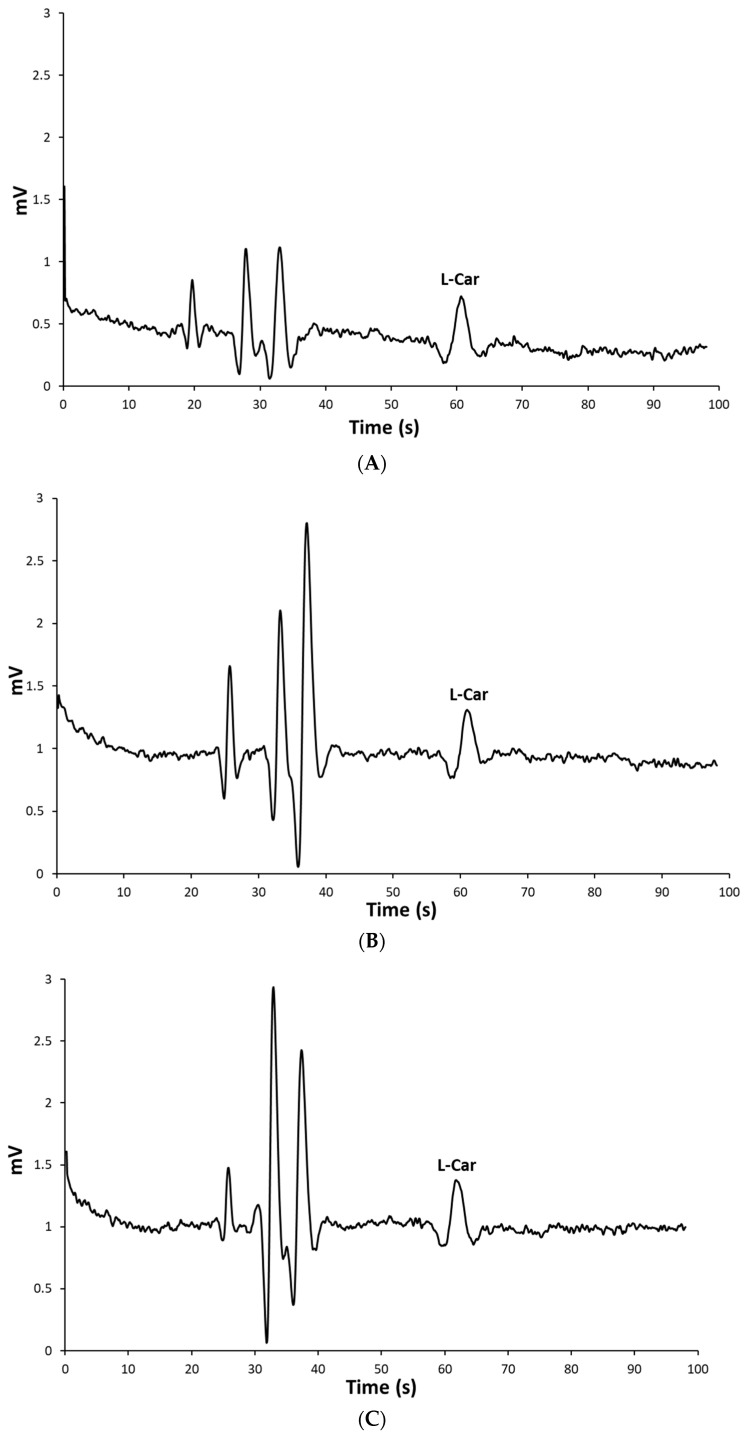

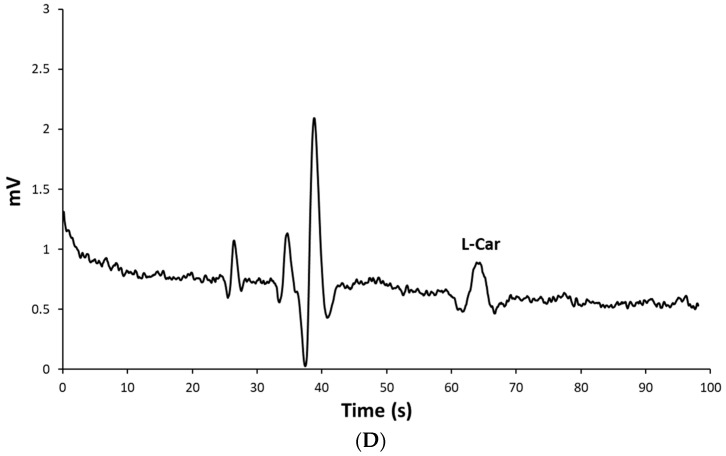
MCE-C^4^D electropherogram for L-carnosine (**A**) 5 × 10^−5^ M standard solutions, (**B**) Sample 1, (**C**) Sample 2, and (**D**) Sample 3. Used conditions: BGE consisted of 0.5 mol L^−1^ (pH 2.52), 20 s electrokinetic injection, and +2.75 kV applied separation voltage.

The developed electrophoretic method applicability was demonstrated using the selected experimental protocols and instrumental parameters for the quantification of L-carnosine in three different multicomponent health supplements. The procedure did not involve any sample preconcentration or derivatization of the analyte which are commonly used in peptide analysis. The electropherograms of the samples are shown in Figure 3. In all three electropherograms, the peaks corresponding to L-carnosine are well-defined, symmetrical, and completely separated from the other sample components. It should be noted that the migration times between L-carnosine in the case of the standard solution and those of the sample’s solutions did not differ notably, which indicates the acceptable specificity of the method. The recorded values were 61 ± 0.42 s, 61.88 ± 0.59 s, 62.28 ± 0.6 s, and 63.35 ± 1.2 s for the standard solution, Sample 1, Sample 2, and Sample 3, respectively. The method specificity was further confirmed by spiking the samples with L-carnosine standard solutions.

The results summarized in Table 2 show that all three samples had a slightly higher content of L-carnosine than declared. These minor differences may be attributed to the capsule form of the supplement, as the highest deviations were observed in the capsule samples and the lowest in the powder samples [28]. In this study, the L-carnosine concentrations obtained were 106.03–115.90% of the reported value, which was within the tolerance variation. The highest variation was observed in Sample 3, which contained a concentration of L-carnosine five times higher than the other two samples. Sample 1 and Sample 2 contained a similar declared value for L-carnosine, so the deviations from the declared value were also similar: +7.65 and +6.03 for Sample 1 and Sample 2, respectively, raising the suspicion that the deviation from the declared values increases with an increasing amount of the active ingredient content. However, more samples are needed to confirm these claims. For the time being, however, the focus of the study was on developing an accurate and precise method for evaluating L-carnosine content.

Recovery experiments were performed with two different concentrations to confirm the accuracy of the method and to exclude the influence of the matrix on L-carnosine analysis. Each sample was spiked with L-carnosine to obtain concentrations 20% and 40% higher than those already detected. The recovery values ranged from 94.46 to 112% and detailed information is given in Table 3. As another approach to assessing the method’s accuracy, CE-UV-VIS and an HPLC-DAD were employed for further analysis of the samples. Table 2 shows the comparable results for L-carnosine concentration with all the methods used. By applying the *t*-test (n = 3, α = 0.05, t_crit_ = 4.303), it was found that there were no statistically significant differences between the precision of the concentrations of MCE-C^4^D and CE-UV-VIS. The calculated t-values were 0.70, 2.05, and 0.52 for Samples 1, 2, and 3, respectively. However, the t-test did not show statistically significant differences, even when comparing the proposed method and HPLC-DAD. The calculated t-values were 3.1620, 0.5424, and 0.1835 for Samples 1, 2, and 3, respectively. A very good correlation between these methods indicates a reasonable accuracy of the developed method for the determination of L-carnosine in health supplements.

**Figure 4:**
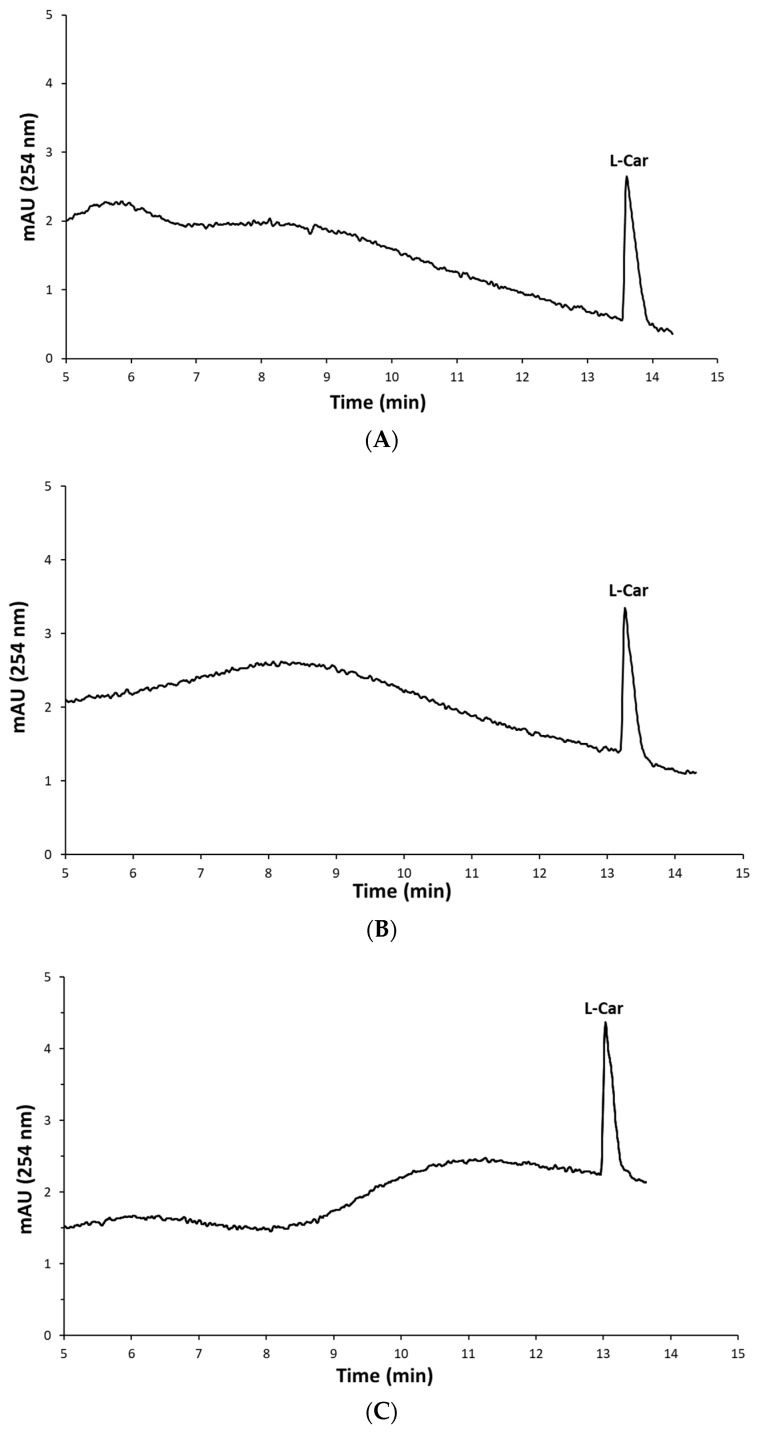
CE-UV-VIS electropherogram of (**A**) Sample 1, (**B**) Sample 2, and (**C**) Sample 3. CE conditions: 50 mM CuSO_4_ + 0.05% HAc electrophoretic buffer (pH 4.5), hydrodynamic injection at 50 mbar for 5 s, and +15 kV separation voltage.

A comparison of the analytical characteristics of all methods used is presented in Table 4. It is noteworthy that the coefficient of determination was higher in the case of the MCE-C^4^D analysis than in the CE-UV-VIS analysis. Furthermore, observing the CE electropherograms of the samples (Figure 4), it is evident that the analysis time was significantly longer using CE-UV-VIS than MCE-C^4^D. The recorded retention time of L-carnosine on the CE was 807.72 ± 2.49 s, while the migration time on the MCE was only 61 ± 0.42 s.

A lot of the available literature deals with the MCE analysis of proteins, polypeptides, and their digestion products, still, fewer papers describe the MCE analysis of small peptides, especially dipeptides. An overview of the MCE methods for the analysis of peptides containing less than 20 AAs residues is presented in Table 5. To date, a few methods, each of similar sensitivity, have been reported on the MCE of dipeptides. As in this study, micromolar concentrations were analyzed, however, slightly lower LODs were achieved using optical detectors, which is expected. For example, reported LODs for the chemiluminescence detection of L-carnosine, L-homocarnosine, and L-anserine were 0.030 μM, 0.028 μM, and 0.034 μM, respectively [21], while laser-induced fluorescence (LIF) detection was performed with LODs of 0.050 μM for Gly-Gly and Gly-Leu [29], and 0.065 μM for L-carnosine [22]. MCE was utilized in the CE-fluorescence analysis as a preconcentration method for Phe-Ala, which enabled for concentration enrichment from 2 μM to 0.02 μM [30]. Considering the linear detection range of our method was sufficient for the analysis of food supplements, and it is assumed to be appropriate for the other types of samples, it can be said that the slightly higher detection limit of 2.5 μM is a reasonable compensation for avoiding expensive instrumentation and the use of derivatization agents. Studies using a homemade MCE-C^4^D unit reached somewhat better sensitivity; LODs were below 1 μM for L-carnosine and L-anserine [26,27]. However, in this case, complex samples’ pre-concentration was necessary since the meat samples contain a large amount of proteins and fats. Unlike for the HPLC analysis of meat samples, the prepared samples’ aliquots needed to be further deproteinized and concentrated, which included several extra steps: dilution by methanol, shaking, storing at 4 °C, centrifugation, filtration, and supernatant evaporation to dryness. The solid residues were then supplemented with an extremely small water volume, shaken, heated, and finally sonicated. In contrast, in this study, method simplicity and rapidness are emphasized. MCE-C^4^D analysis of the L-carnosine in capsule samples required only two simple steps since the concentration was significantly higher than those in meat samples. Therefore, there was no demand for deproteinization, sample enrichment, or extensive BGE optimization in order to lower the detection limit. This indicates the huge potential of MCE-C^4^D for the analysis of samples such as health supplements, dietary supplements, or different medicaments.

## 3. Materials and Methods

### 3.1. Chemicals and Materials

Acetic acid (Panreac Quimica Ltd., Barcelona, Spain), citric acid (Sigma-Aldrich, Saint Louis, MO, USA), glycolic acid (Sigma-Aldrich, Saint Louis, MO, USA), iminodiacetic acid (Sigma-Aldrich, Saint Louis, MO, USA), tartaric acid (Sigma-Aldrich, Saint Louis, MO, USA), rubidium acetate (Sigma-Aldrich, Saint Louis, MO, USA), sodium acetate (Sigma-Aldrich, Saint Louis, MO, USA), and 2-hydroxyisobutyric acid (Sigma-Aldrich, Saint Louis, MO, USA) were tested as background electrolyte (BGE) components. L-carnosine of analytical grade (Sigma-Aldrich, Saint Louis, MO, USA) was used for standard solutions. Copper (II) sulfate (T.T.T. Ltd., Novaki, Croatia) was used in UV-VIS detection. All chemicals were of analytical reagent grade. The health supplements were obtained from web shops. They were all in the form of capsules. The declared mass of the capsule in Sample 1 was 583 mg and the mass of L-carnosine was 100 mg. The Sample 2 declaration noted capsules of 361.45 mg of mass that contained 62.35 mg of L-carnosine and 84 mg of zinc. The declared mass of Sample 3 capsules was 600 mg with 500 mg of L-carnosine. The capsules contained various additional ingredients; chromium, calcium carbonate, silicon dioxide, talc, magnesium stearate, and hydroxypropyl methylcellulose. In addition, the samples contained complex mixtures of unknown ingredients due to the dry extract of Chinese cinnamon and rice powder. Before analysis, cellulose syringe filters (0.22 μm pore diameter, Labex Ltd., Marrickville, NSW, Australia) were used to filter the sample solutions.

### 3.2. Real Samples Preparation for MCE–C^4^D Analysis

The sample preparation procedure was identical for all three supplements but differed in dilution. The powder was poured from 10 capsules and crushed in a mortar. Then, 100 mg of the homogenized powders was diluted in 10 mL of ultrapure water (Veolia Water Technologies, Paris, France). The prepared solutions were further diluted. The dilution factors depended on the L-carnosine mass fraction needed to obtain an appropriate concentration of the sample solution. In the case of Sample 1 and Sample 2, the dilution factor was 1:750, and in the case of Sample 3 dilution was 1:5000. Before the analysis, the solutions were filtered using CHROMAFIL Xtra RC-20/25 syringe filters with a pore size of 0.2 μm (Macherey-Nagel GmbH and Co. KG., North Rhine-Westphalia, Germany) and placed in an ultrasonic bath for 10 min before analysis.

### 3.3. MCE–C^4^D Analysis

**Figure 5:**
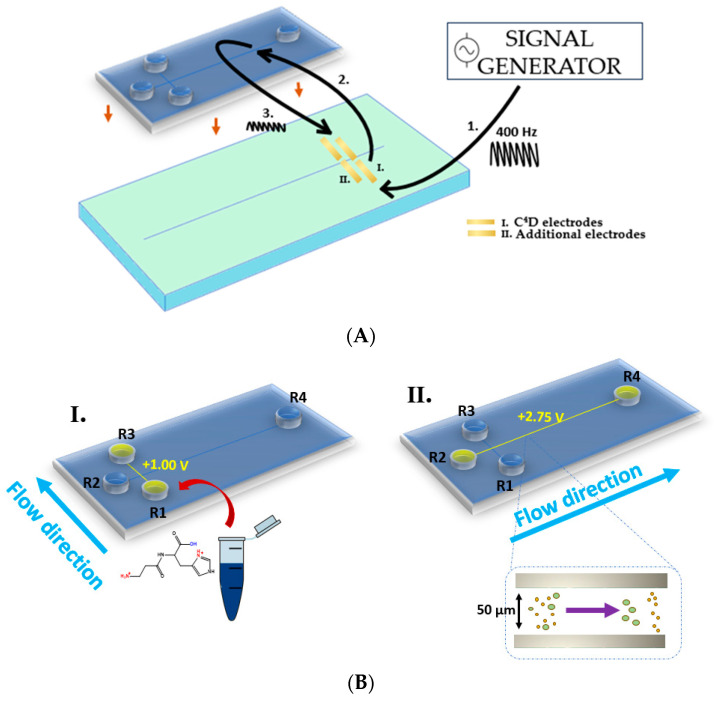
Layout of the assay design for (**A**) integrated MCE-C^4^D analysis, (**B**) MCE sample injection (I.) and separation processes (II.)

**Figure 6:**
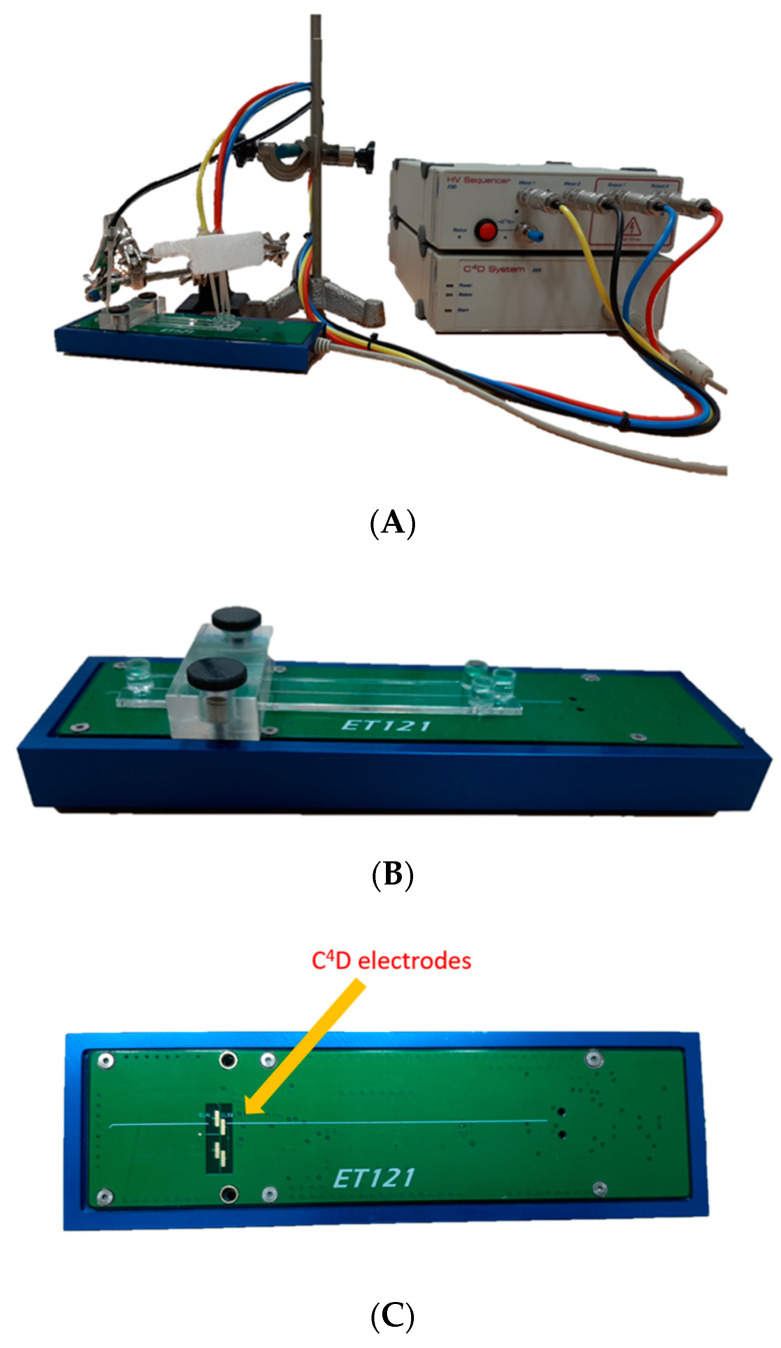
(**A**) Integrated instrumentation of ET121 microchip platform, ER430 High Voltage Sequencer, and ER225 C^4^D system; (**B**) a double-T poly(methyl methacrylate) chip on the ET121 microchip platform; (**C**) display of ET121 microchip platform with C^4^D electrodes.

MCE analyses were performed on a double-T poly(methyl methacrylate) chip (ChipShop GmbH). The dimensions of the chip were 1.6 cm × 9.5 cm. The separation channel had a full length of 8.7 cm and a cross-section of 50 × 50 µm. The chip was placed on the MCE model ET121 (eDAQ) device (Figure 5B), which is connected to an ER430 High Voltage Sequencer (HVS) and an ER225 C^4^D system (Figure 6A). The detection electrodes are located at the ET121 platform, separated from the microchip. Indeed, the ET121 platform contained four 2 mm × 1 mm gold electrodes (Figure 6C and Figure 5A), two of which are used for detection. Before the measurement, the microchip was positioned on the platform in such a manner that the electrodes were located at the end of the separation microchannel. The electrodes touched only the microchip outside and were still separated from the microchannel and BGE. A high-frequency alternating current was applied on the transmitter electrode, and thereupon was capacitively coupled into the electrolyte and then in the receiver electrode (see Figure 5A). The signal decreased due to the passing through the BGE and the size of the received signal depended on the electrical conductivity of the sample. Due to the coupling of the detection electrodes, stray capacitance would be generated, however, in a four-electrode configuration it was efficiently eliminated. The pairing of the other two electrodes also produced a stray capacitance that annulled the stray capacitance created by the first pair of electrodes. In this way, the negative influence of stray capacitance on the signal was significantly reduced. Preparation of the chip for analysis involved a simple rinse of the microchannel, first with ultrapure water and then with BGE. To test the current flow and gauge the stability of the measuring device and microchannels permeability, 50 µL of BGE and high voltage electrodes were added to each chip reservoir and a blank test was performed. If the baseline was suitable, a sample was recorded. The sample solution was placed in reservoir 1 (R1) and BGE in reservoirs 2, 3, and 4 (R2, R3, R4). Only electrodes 1 and 3 were controlled, during floating injection by applying 1000 mV on R1 and ground on R3 for 20 s (illustrated in Figure 5B(I.)). The resulting current was 40 μA due to microchannels double-T geometry, in which the distance between R1 and R3 was only 1 cm. This injection microchannel crossed over an 8.7 cm length separation channel at 0.5 cm of length. At 21 s, the sample was at the intersection of the two microchannels and the separation was started. At this moment the electrodes in R1 and R3 were disconnected from the power supply and 2750 mV was applied to R2 and ground on R4 for 260 s (illustrated in Figure 5B(II.)). Thus, the sample migrated to R4, and just before reaching it, the sample was detected on the C^4^D detector placed on the MCE platform (outside the microchip). A signal was detected using an excitation sine wave of 400 kHz with an amplitude of 40%. Each sample was recorded in three series, with one series comprising five consecutive measurements. Between each series of measurements, the microchannels were flushed with BGE in order to achieve satisfactory reproducibility. Upon the end of the experiments, the microchannels were rinsed with ultrapure water. The obtained C^4^D electropherograms were analyzed using PowerChrom (eDAQ, Version 2.7.8, Denistone East, NSW, Australia). In the used high-conductivity acidic BGE, the obtained signals were negative and therefore inverted.

### 3.4. CE-UV-VIS Analysis

Agilent Technologies 7100 CE instrument (Waldbronn, Germany) and fused-silica capillary (Polymicro Technologies, Phoenix, AZ, USA) with an effective length of 41.6 cm × 50 μm ID were used for CE analysis. The 3D-CE ChemStation software, Agilent Technologies (B. 04.00. and Version 7.01, Waldbronn, Germany) was used for UV detection data processing. Measurements were performed using a running buffer consisting of 5 × 10^−5^ M CuSO_4_ and 0.05% HAc (pH 4.5) as previously reported in the work of Jiang et al. [44]. Direct analysis of the underivatized L-carnosine was possible due to the complex formation between Cu^2+^ and L-carnosine which absorbs at a wavelength of 254 nm.

In this work, the optimized hydrodynamic injection at 50 mbar for 5 s was applied. The electrophoretic separations were performed with a voltage of +15.00 kV. Before analysis, the capillary was rinsed with aqueous solutions in the following order: (1) ultrapure water for 5 min; (2) 0.1 M NaOH for 5 min; (3) ultrapure water for 5 min; (4) running buffer for 5 min. Between each run, the capillary was rinsed with a running buffer for 5 min. At the end of each day, the capillary was rinsed in the following order: (1) ultrapure water for 5 min; (2) 0.1 M NaOH for 5 min; (3) ultrapure water for 10 min.

### 3.5. HPLC-DAD Analysis

Additionally, method evaluation was performed using HPLC-DAD analysis, according to the official amino acid analysis methodology of Rita Steed [45]. The used instrumentation was an Agilent 1260 Infinity II HPLC system (Agilent, Santa Clara, CA, USA) containing a quaternary pump system, autosampler, and column department. For the acquisition and processing of the data, Agilent OpenLAB CDS software (Version 2.6, Agilent, Santa Clara, CA, USA) was employed. The separations were performed on the Poroshell 120 EC-C18, at 2.7 μm, 4.6 × 100 mm, sustained at 40 °C (exit side at 35 °C). Before the measurement, all the samples were derivatized with phthalaldehyde for 2 min. Two different mobile phases were used for gradient elution; MP A (10 mM Na_2_HPO_4_–10 mM Na_2_B_4_O_7_, pH 8.2) and MP B (45 ACN:45 MeOH:10 H_2_O by volume). The ratio of mobile phase B changed as follows: 2% in the start time, 2.00% in 1 min, 57.00% in 7 min, 100.00% in 7.1 min, 100.00% in 8.4 min, and 2.00% in 8.6 min. In 0.00 min, the L-carnosine was measured at bandwidths 338 nm and 10 nm (Ref 390 nm and 20 nm). Thereafter, it was measured in 5.53 min at bandwidths 262 and 16 nm (Ref 324 and 8 nm).

## 4. Conclusions

A simple electrophoretic method for the determination of L-carnosine with a short analysis time was developed using the integrated system of a C^4^D detector and microchip platform. The main advantages of the presented method are its low price and simple operating procedure without a derivatization process. Analytical parameters such as LOD, LOQ, linear range, RDS, and recovery values confirmed the suitability of the proposed method for the analysis of health supplements containing L-carnosine. The accuracy of the method was confirmed by the standard addition method and the comparative sample analyses at CE-UV and HPLC-DAD. Thus, this study supports the potential of using MCE-C^4^D methods for quality control in the health supplement industry.

## Figures and Tables

**Table 1:** Analytical characteristics of the MCE-C^4^D methods.

Characteristics	MCE-C^4^D
Migration time (s)	61 ± 0.42
R^2^	0.9976
Linear range (M)	5 × 10^−6^ – 5 × 10^−5^
Slope	27,567
Intercept *	0.1852
LOD (μM)	2.5 × 10^−6^
LOQ (μM)	5 × 10^−6^

* Mean ± standard deviations (n = 3). Abbreviations: LOD, limit of detection, LOD; limit of quantification, R^2^, coefficient of determination.

**Table 2:** Results of the quantitative determinations of L-carnosine in health supplement samples.

		Sample 1	Sample 2	Sample 3
	Declared Value (mg/g Sample)	171.42	172.50	833.33
MCE-C^4^D analysis	Results (mg/g sample)	184.53 ± 8.28	182.91 ± 15.95	965.79 ± 130.50
	RSD (%)	4.49	8.72	13.51
	% Deviation from the declared value	+7.65 ± 4.83	+6.03 ± 9.24	+15.95 ± 15.66
CE-UV-VIS analysis	Results (mg/g sample)	178.60 ± 6.39	167.7 ± 4.57	927.72 ± 3.45
	RSD (%)	3.58	2.72	0.37
	% Deviation from the declared value	+4.19 ± 3.73	−2.78 ± 2.65	+11.33 ± 0.42
HPLC-DAD analysis	Results (mg/g sample)	198.01 ± 9.80	177.4 ± 8.48	980.63 ± 47.10
	RSD (%)	5	5	5
	% Deviation from the declared value	+15.51 ± 5.71	+2.84 ± 4.92	+17.68 ± 5.65

**Table 3:** MCE-C^4^D results of recovery experiment for spiked health supplement sample.

	Unspiked Sample (μM)	Added Amount (μM)	Spiked Sample (μM)	Recovery (%)
	* Recovery experiment 1			
Sample 1	11.01 ± 0.04	2.20	13.09 ± 0.07	96.66 ± 1.90
Sample 2	10.19 ± 0.10	2.18	13.09 ± 0.07	99.73 ± 3.32
Sample 3	8.53 ± 0.010	1.70	10.28 ± 0.08	102.36 ± 4.91
	* Recovery experiment 2			
Sample 1	11.01 ± 0.04	4.40	15.76 ± 0.20	108.00 ± 4.54
Sample 2	10.19 ± 0.10	4.36	15.22 ± 0.23	98.62 ± 5.29
Sample 3	8.53 ± 0.010	3.41	12.12 ± 0.25	108.73 ± 3.20

Recovery experiment 1—L-carnosine was added to obtain a 20% higher value than an unspiked sample. Recovery experiment 2—L-carnosine was added to obtain a 40% higher value than an unspiked sample * Number of measurements = 3.

**Table 4:** Analytical characteristics of the MCE-C^4^D, CE-UV-VIS, and HPLC-DAD methods.

Characteristics	ME-C^4^D	CE-UV-VIS	HPLC-DAD
R^2^	0.9976	0.9905	0.9996
Measured concentration range	5 × 10^−6^–5 × 10^−5^	4.4 × 10^−4^–4.4 × 10^−3^	5 × 10^−6^–2.5 × 10^−4^
Slope	27,567	64.022	3.72 × 10^−7^
Intercept *	0.1852	−3.938	2.43 × 10^−6^

* Mean ± standard deviations (n = 3).

**Table 5:** An overview of the MCE methods for the analysis of oligopeptides containing less than 20 AAs residues.

Analyte/Sample	BGE	Chip Material	Linear Range/Tested Range	LOD (Analyzed Conc.) (μM)	Detector	Ref.
βA-H (carnosine)/health supplements	0.5 M HAc	PMMA	5–50	2.5	C^4^D	This work
βA-H (carnosine), homocarnosine (GABA-H) anserine (βA-methyl-H)/human cerebrospinal fluid and canine plasma	15 mM borate buffer (pH 9.8) + 1.0 mM Co^2+^ + 1.0 mM adenine +35 mM SDS	glass/PDMS	0.06–15	0.030 0.028 0.034	CL	[21]
βA-H (carnosine) βA-methyl-H (anserine)/breast muscle	200 mM HAc + 10 mM HIBA + 0.3 mM KOAc (pH 2.7)	PMMA	0–200	0.10 0.16	C^4^D	[26]
βA-H (carnosine)/macrophage cells	20 mM borate (pH 9.2)	glass	0.025–5	0.065	LIF	[22]
βA-H (carnosine), βA-methyl-H (anserine)/meat	250 Mm HAc + 10 mM 2-OH-n-butyric acid + 0.3 mM RbOAc (pH 2.6)	PMMA	0–88.42	0.038 0.069	C^4^D	[27]
βA-H (carnosine)/cosmetics	0.1 mM borax with + 0.03% *w*/*w* SDS	BSA- COC	0.38–226.24 mg/kg	0.17 mg/kg, (0.10, 10)	LIF	[23]
GG, GL, RPP, KPV, VKK WYD, YWS/ anode chamber of microbial fuel	10 mM borax-H_3_BO_3_ buffer + 20 mM SDS (pH = 9.0)	ITO-glass	0.625–10 0.625–10 0.31–10 1.25–20 1.25–20 0.625–10 0.625–10	0.050 0.050 0.025 0.625 0.100 0.050 0.050	LIF	[29]
GF isomers/ model solution	20 mM PBS (pH 7.17)	PDA/ GO/BSA-PDMS	N/A	N/A (4000)	Amperometry	[31]
FA, YGGFL (leucine enkephalin), GGYR/model solution	10 mM Na_2_CO_3_ + 0.5% HPC (*w*/*v*) (pH 9.7)	HPC-PDMS	N/A 1–10 N/A	N/A (0.02)	Fluorescence	[30]
GGGG GGGGG GGGGGG/model solution	20 mM Na_2_B_4_O_7_ (pH 10.02)	glass	N/A	N/A (10)	Fluorescence	[32]
GCE (glutathione)/ rat liver cells	20 mM Na_3_PO_4_ + 1.5 mM luminol (pH 9.6)	glass	3–600	0.96	CL	[33]
XLYENKPRRPYIL (neurote-nsin), CYIQNCPLG (oxytocin), YGGFL (leucine enkephalin), EADPNK + FYGLM (tryptic digestion of physalaemin)/model solution	Ampholyte mixture (pH 4–7)	acrylate-glass	N/A	N/A (100, 100, 100, 966)	Fluorescence	[34]
pI markers: GCDDD (pI 3.64), GCHQHQHQHQ (pI 53) * GCQHHHR (pI 7.58) * GCYYYKK (pI 9.56)/model solution	2.5% (*v*/*v*) Pharmalyte 3–10	glass	N/A	3 × 10^−6^ for the pI 5.53 (0.0025)	LIF	[35]
β-casomorphins: YPFPG, TPFPGP, YPFPGPI, TPFPG, PFPGPI/cheese	15 mM H_3_BO_3_- Na_2_H_20_B_4_O_17_ (pH 10.5)	glass	0.01–1 0.05–1 0.05–1 0.03–1 0.05–1	0.075 0.042 0.024 0.019 0.019	LIF	[36]
DRVYIHPFHL (Angiotensin I), DRVYIHPF (Angiotensin II), RVYIHPF (Angiotensin III), RPPGF (Bradykinin fragment 1–5)/model solution	30 mM NH_4_Ac + 30% MeOH	PDMS	N/A	N/A (100)	ESI/MS	[37]
RPPGFSP (Bradykinin fragment 1–7), SYSMEHFRW, GKPVGKKR (ACTH fragment 1–17)/model solution	ACN: 10 mmol/L Tris buffer (pH 8.2)/80:20	NPs -silicon	N/A	N/A (6)	MALDI-TOF-MS	[38]
Vancomycin (7 modified AAs); (NLEU)-(HDPY)-(ASN)–p-(DPYG)-(DHPG)-HDPY-(LPGH)/human plasma	300 mM HAc	glass	5.18–4.86	0.83	C^4^D	[39]
Ac-R-[CMLNRVYRPC]/model solution	25 mM phosphate buffer (pH 7)	PEG- glass	N/A	N/A (100)	Fluorescence	[40]
PO_4_-glycogen synthase (PLSRTLSVSS)/model solution	100 mM Tris phosphate buffer (pH 7.0)/10 mM EDTA	PMMA	0.001–0.025	0.001	Fluorescence	[41]
PO_4_-peptides: DHTGFLpTEYVATR, DHTGFLTEpYVATR, DHTGFLpTEpYVATR, Non-PO_4_-peptide: DHTGFLTEYVATR	25 mM borax + 2.8% NH_4_OH (pH 11.5)	glass	0.04–1.34	0.015 for PO_4_-peptides (0.31)	Fluorescence	[42]
Insulin receptor peptide: Non-PO_4_-peptides: TRDIYETDYYRK PO_4_-peptides: TRDIpYETDYYRK TRDIYETDpYYRK TRDIpYETDpYpYRK	40 H_2_O/60 MeOH (*v*/*v*) + 30 mM NH_4_-Ac (pH 7.4)	PDMS	N/A	N/A (125, 250)	ESI-MS	[43]

Abbreviations: Ac-R-[CMLNRVYRPC], Acetyl-D-Arg-[Cys-Met-Leu-Asn-ArgVal-Tyr-Arg-Pro-Cys]-NH_2_; BS, bovine serum albumin; CL, chemiluminescence; COC, olefin copolymermicrochips; ESI-MS; electrospray ionization-mass spectrometry; GABA, Gamma-aminobutyric acid; HAc, acetic acid; HPC, Hydroxypropyl cellulose; ITO, indium tin oxide; MALDI-TOF-MS, matrix assisted laser desorption ionization-time of flight mass spectrometry; (NLEU)-(HDPY)-(ASN)–p-(DPYG)-(DHPG)-HDPY-(LPGH), N-methyl-D-leucine-m-chloro-bhydroxy-D-tyrosine-asparagine-p-(2-[a4-L-epi-vancosaminyl]-b-1-D-glucosyl)-D-phenylglycine–p-hydroxy-D-phenylglycine-HDPY-m,m-dihydroxy-L phenyl-glycine; Non-PO_4_-peptides, nonphosphorylated peptides; (NPs, nanoparticles; PDA/GO/BSA; bovine serum albumin conjugated polydopamine–graphene oxide nanocomposites, PO_4_-peptides,phosphorylated; SDS, sodium dodecyl sulfate.

## Data Availability

The data presented in this study are available by contacting the authors.
